# The functional and molecular impact of triamcinolone acetonide on primary human bone marrow mesenchymal stem cells

**DOI:** 10.1038/s41598-023-48448-z

**Published:** 2023-12-08

**Authors:** Maritha Kumlin, Johanna Ungerstedt, Huan Cai, Elory Leonard, Li Felländer-Tsai, Hong Qian

**Affiliations:** 1 Division of Orthopaedics and Biotechnology, Department of Clinical Science, Intervention and Technology (CLINTEC), Karolinska Institutet, K54, 141 86 Stockholm, Sweden; 2 Department of Medicine Huddinge, Center for Hematology and Regenerative Medicine (HERM), Karolinska Institute, Stockholm, Sweden; 3 ME Hematology, Karolinska University Hospital, Stockholm, Sweden

**Keywords:** Mesenchymal stem cells, Stem-cell differentiation

## Abstract

Traumatic or degenerative joint pain is abundant in the population. Symptom relief by intra- and periarticular glucocorticoid administration is frequently used, however may have potentially devastating effects, changing the normal healing process of the joint. Mesenchymal stem cells (MSCs) are important for wound-healing processes due to their multipotency in regenerating osteoblasts, chondrocytes and adipocytes but also have immunomodulatory properties. The aim of this study was to investigate the impact of triamcinolone acetonide (TA) a common glucocorticoid administrated intra- and periarticularly, on human bone marrow derived MSC viability, functionality, multi-lineage differentiation and transcriptomic output. We found that TA treatment induced apoptosis and promoted adipogenesis while impairing chondrogenesis of MSCs. RNA sequencing indicated that TA modulated the inflammatory response of MSCs, which may have an impact on the immunologic environment where the inflammatory phase is a physiological part of the natural healing process. These data indicate that triamcinolone acetonide should be used with consideration bearing the patient’s outcome in mind, with the intention to optimize joint recovery and homeostasis.

## Introduction

During the last decades, there has been considerable focus on optimizing the treatment of joint pain and fracture healing^1^. Injuries to tendons, as well as fractures, are common in the ageing population as well as in younger patients. The healing phases remain similar regardless of age, starting with the inflammatory phase, followed by the proliferative phase and remodeling phase, which all together can last for more than a year^2^. Disturbances in tissue homeostasis in any of the phases may lead to impaired or prolonged healing process which often is difficult to reverse.

Mesenchymal stem cell (MSC) and/or growth factors have been investigated as cell-based therapy due to their regenerative and differentiating capacity into mesenchymal lineages including adipocytes, bone-forming osteoblasts and chondrocytes forming fat, bone and cartilage, respectively^3–8^, as well as their immunomodulating properties^9^. Any potential adverse impact of the pain-relieving pharmacological treatment on MSC maintenance and differentiation may influence the healing process. Traditional treatments with anti-inflammatory and pain-relieving drugs have been the standard symptom treatment for this patient group with little or no consideration of potential long-term adverse effects^10^. The current state-of-the art-treatment is far from a precision-based therapy where the individual patient´s healing process and treatment response have been taken into consideration.

Among the drugs frequently used for pain relief after joint injury, triamcinolone acetonide (TA) and diclofenac have been studied in in vitro animal models^11–13^. Studies on bone marrow (BM) derived human MSCs have shown that TA induces impaired MSC growth and promoted adipocyte infiltration deteriorating healing and recovery^14–16^. However, the molecular mechanisms and functional consequences of short- or long-term TA treatment in MSC regeneration and differentiation are not well understood.

This study aims at assessing the effects of TA on the maintenance, expansion and differentiation of human BM MSCs under hypoxic conditions mimicking the in vivo situation, as well as the MSC transcriptomic output. We found that short-term exposure of MSCs to 10 nM TA ex vivo impaired MSC viability, colony-forming unit capacity (CFU-F), promoted adipocyte differentiation, compromised chondrocyte differentiation, and functionally altered inflammatory response genes. The findings may raise a concern about long-term treatment with TA particularly for patients with underlying chronic diseases associated with impaired wound an tissue healing.

## Methods

### Subjects

Iliac crest BM aspirates were retrieved from four healthy donors after oral and written informed consent. The study was performed according to the Helsinki declaration and approval by the regional ethics committee (No 2008/364-31).

### Bone marrow derived MSC cultures

Mononuclear cells from BM aspirates were isolated by gradient density centrifugation with Ficoll-Hypaque (LymphoPrep, Axis Shield Poc As). Cells were cultured in plastic tissue culture flasks under hypoxic conditions 1% O_2_ in complete DMEM (DMEM high glucose 4.5 g/L, Gibco, Carlsbad, CA USA, supplemented with 10% Fetal Bovine Serum (Gibco, Carlsbad, CA USA), 1% penicillin/streptomycin (Hyclone, Logan, UT, USA), 10 mM HEPES Buffer (R&D Systems, Minneapolis, MN, USA), 10^−4^ M 2-mercapthoethanol (Sigma-Aaldrich, Darmstadt, Germany). The media was changed every 3 days to remove hematopoietic cells. The plastic adherent cells were dissociated and serially passaged at 80% confluence. The culture-selected MSCs at passage 1–5 were used for experiments.

### MSC surface marker analysis by flow cytometry

Cells at passage 1–5 were collected for cell surface marker expression analysis. This includes negative markers including CD45 and CD31, and positive markers including CD44, CD105 and CD90. The cells were stained with anti-human CD45-BV421, CD31-PECY7, CD44-APC-CY7, CD105-APC and CD90-PECY5 (Nordic Biosite) (Supplementary Table 2). Subsequently, the cells were stained with PI (0.5 ug/mL) 5 min prior to flow cytometry analysis on Fortessa (BD). Data analysis was carried out using FlowJo software (TreeStar Inc).

### Cell growth and viability effects of TA

MSCs at passage 1–5 were plated at 2500 cells/cm^2^ and let to adhere overnight. The cells were then subjected to 1 nM–1 µM TA (Sigma-Aldrich, Darmstadt, Germany) in complete DMEM or to complete DMEM only as control. DMSO in 5% was used as a positive control for induced cell death. Cells were collected at 4 h, 24 h and 48 h and manually counted in a Burker chamber using 0.08% Trypan blue solution. The experiment was performed in duplicates and was repeated three times. IC50 was assessed at 24 h and 48 h. Cell viability and apoptosis was assessed at 24 h and 48 h when the cells were collected and stained with Annexin V (AV) and Propidium Iodide (PI) (Invitrogen, Waltham, MA, USA) and assessed by flow cytometry using Fortessa (BD, bioscience). The data was analyzed using FlowJo software (TreeStar Inc).

### Colony forming units in fibroblasts (CFU-F) assay

CFU-F assessing MSC self-renewal and their functionality was performed as described^17,18^. Briefly, the cells were first exposed to complete DMEM or complete DMEM + 10 nM TA for 24 h. After washing, cells were seeded at 200 cells/well in a 6 -well plastic plate and cultured under hypoxic conditions (1% O_2_) for 10–12 days, fixed in methanol and stained with Giemsa (Sigma-Aaldrich, Darmstadt, Germany) for CFU-F colony counting. Experiments were performed in duplicates and repeated three times. Pictures were taken under inverted microscope (CKX41, Olympus).

### Multilineage differentiation assay

After 24 h incubation of BM MSCs at passage 1–5 with or without 10 nM TA under hypoxic conditions, cells were dissociated and collected for the respective differentiation assay, as previously described^17^.

In brief, for adipocyte differentiation cells were incubated 2–3 weeks in normoxia with 20% O_2_, in complete DMEM supplemented with 5 ug/ml insulin (Sigma-Aaldrich, Darmstadt, Germany), 10^−6^ M dexamethasone (Sigma-Aaldrich, Darmstadt, Germany), 20 µM indomethacin (Sigma-Aaldrich, Darmstadt, Germany) and 0.0115 mg/ml of IBMX (Sigma-Aaldrich, Darmstadt, Germany) and the control undifferentiated cells were subjected to complete DMEM with 10% FBS. Cell media were changed every 2–3 days. After 2–3 weeks, cells were fixed in 10% formalin and then washed with 60% isopropanol. Adipogenic differentiation was evaluated with oil-red-O staining (Sigma-Aaldrich, Darmstadt, Germany). Pictures were taken using an Olympus phase contrast camera. To quantify differentiated adipocytes with oil red O staining, the oil red O staining was eluted with 100% isopropanol for 15 min. Optical density (OD) was measured on an Tecan Infinite Pro 200 apparatus using Magellan software at 450 nM. For osteogenic differentiation, cells were cultured in osteogenic differentiation media (CCM008, R&D Systems, Minneapolis, MN, USA) and the control (undifferentiated) was maintained in complete DMEM medium (for 3 weeks under normoxic conditions with 20% 0_2_. Cells were fixed in ice cold methanol and stained with 2% Alizarine red (Sigma-Aaldrich, Darmstadt, Germany) to verify calcium deposits. Pictures were taken using an Olympus bright field camera. Alizarin staining was eluted with 10% cetylpyridinium chloride for 1 h. Eluation was quantified on an Tecan Infinite Pro 200 apparatus using Magellan software at 570 nM.

### In vitro chondrocytic differentiation in 3D pellet culture

For in vitro chondrocytic differentiation in 3D pellet culture, 250 000 cells were harvested, spun down and dissolved in 500 μM of DMEM high glucose with 10 mM HEPES, 100U of penicillin/streptomycin, 10^−4^ M 2-Mercaptoethanol (M7522, Sigma), 2 mM pyruvate (P5280, Sigma), 0.35 mM L-proline (P5607-25G, Sigma), ITS^+3^ (I-3146, Sigma), 5 μg/mL L-ascorbic acid 2-phosphate (A7506, Sigma), 10^−7^ M dexamethasone (D2915, Sigma,), and 10 ng/mL TGF-β3 (100-36E, Peprotech) were added. Cells were cultured at 37 °C under hypoxic condition (1% O_2_) for 28 days, whereafter the micromass pellets were washed with PBS and fixed in 4% PFA. After dehydration with 30% sucrose, the pellets were stained with toluidine blue (T3260, Sigma), washed and embedded in OCT-compound (4583, Sakura Tissue Tek^®^). To visualize the formation of proteoglycan, the embedded pellet was cut to 5 μm, mounted and observed by an inverted microscope (Axio Observer.Z1, Zeiss). Images were processed with Zen software (Carl Zeiss Microscopy, GmbH 2011).

### RNA sequencing

BM MSCs (n = 4) in passage 1–5, were subjected to either TA 10 nM in complete DMEM or in complete DMEM only as control for 24 h, whereafter cells were lysed in RLT buffer (Qiagen, Germantown, MD, USA). RNA extraction was done with RNeasy Mini Kit (Qiagen) according to the manufacturer’s protocol. To construct libraries suitable for Illumina sequencing, the Illumina stranded mRNA prep ligation sample preparation protocol was used with starting concentration between 25 and 1000 ng total RNA. The protocol includes mRNA isolation, cDNA synthesis, ligation of adapters and amplification of indexed libraries. The yield and quality of the amplified libraries was analyzed using Qubit by Thermo Fisher and quality was checked by using Agilent Tapestation. The indexed cDNA libraries were normalized and combined, and the pools were sequenced on the Illumina Nextseq 2000 P3 100 cycle sequencing run, generating 58 base paired end reads with dual index. Basecalling and demultiplexing was performed using CASAVA software with default settings generating Fastq files for further downstream mapping and analysis.

Reads were aligned to a reference built from Ensembl GRCm38 genome sequences using STAR (RRID:SCR_004463, v2.6.1d). All mapped counts to each gene were further calculated by FeatureCounts function from Subread package^19^ installed in R. Genes with Reads Per Kilobase of transcript per Million mapped reads (RPKM) values more than 0.1 were considered as being actively transcribed and proceeded to the analysis of Differential Gene Expression (DGE)^20^. The normalized read counts assigned to each sample were generated by Deseq2 (RRID:SCR_015687). The differentially expressed genes between the cell subsets were identified by adjusted *P* value (*p*adj < 0.05) using Benjamini–Hochberg correction for multiple testing, together with thresholds at log_2_fold changes > 1 (up-regulated) or < -1 (down-regulated). For the Gene Set Enrichment Analysis (GSEA), the normalized read counts were imported into the GSEA (v4.0.3) platform from Broad Institute (http://www.broadinstitute.org/gsea/index.jsp), with three gene sets being tested, including gene ontology, Hallmark and KEGG. Gene sets tested were considered to be statistically enriched when the nominal *P* value < 0.01 and FDR < 0.25. The RNA sequencing data are deposited at Gene Expression Ommibus (GEO) at NCBI website with accession number GSE240387.

### Quantitative RT-PCR (qPCR)

Culture expanded BM MSCs from n = 4 donors, with or without TA treatment, were lysed to RLT buffer (Qiagen) + 2-Mercaptoethanol. RNA extraction was performed with RNeasy Micro Kit (Qiagen) according to the manufacturer’s protocol. Subsequent cDNA synthesizing was carried out using SuperScript IV (Invitrogen) and random primer (Invitrogen). PCR master mix was prepared by mixing 2 × Taqman universal PCR master mix, RNase-free H_2_O, and 20xTaqMan primer–probe mix (Invitrogen). qPCR assay was performed using Biorad C1000 Touch Thermal Cycler instrument. See supplemental Table 3 for information on Assays-on-Demand probes.

### Statistical analysis

Student’s *t* test was used to compare means of two different groups. Non-linear regression was used assessing IC50. All analyses were carried out using the Graph Pad Prism 9.0 system. *P* value < 0.05 was considered statistically significant.

### Data availability

The RNA data sequencing data are available at https://www.ncbi.nlm.nih.gov/geo/query/acc.cgi?acc=GSE240387 with the accession number GSE240387. Remaining data can be provided upon request by the corresponding author.

### Ethical approval

Ethics approved by Regional Ethics Committee in Stockholm (Regionala Etikprövningsnämnden i Stockholm; Meit Camving, chairman; Pierre Lafolie, scientific secretary; Elisabeth Faxelid; Christina Hultman; Anna Kernell; Bernt Lindelöf; Sven Lindskog; Christer Paul; Anette von Rosen; Majvi Andersson; Elisabeth Dingertz; Stig Nyman; Yrsa Stenius; Åsa Öckerman; Pernilla Asp, administrator; No 2008/364-31). All samples were obtained according to the Helsinki declaration.

## Results

### TA treatment inhibits MSC proliferation and induces apoptosis

**Figure 1:**
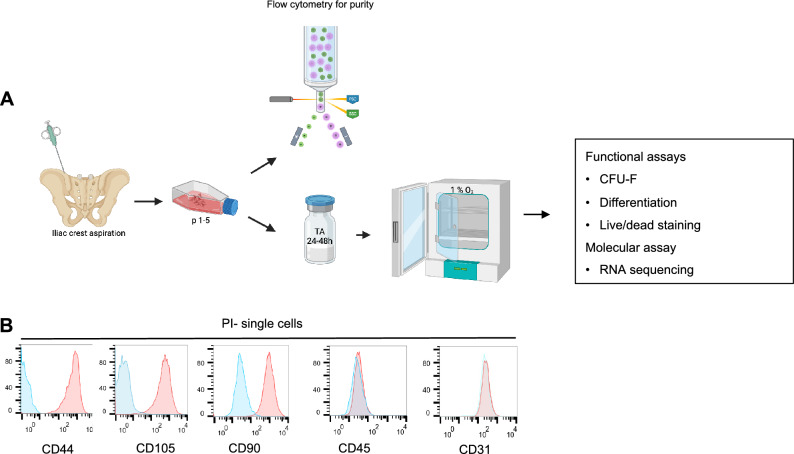
Experimental set up and MSC purity.

To assess the impact of TA treatment on BM MSCs, we isolated MSCs from BM of healthy volunteers and evaluated MSC proliferation, expansion and differentiation by determining viability, colony forming capacity and multilineage differentiation of the cells, as illustrated in Fig. 1A. We first determined purity and identity of the cells by flow cytometry after culture-selection under hypoxic condition by analyzing expression of specific cell surface markers defined by International Society for Cellular Therapy (ISCT)^9^. As expected, these cells at passage 1–5 displayed little or no expression of hematopoietic cell marker (CD45) and endothelial cell marker (CD31) while highly expressing the MSC positive markers including CD44, CD90 and CD105 (Fig. 1B), , suggesting high purity of culture-selected MSCs.

**Figure 2:**
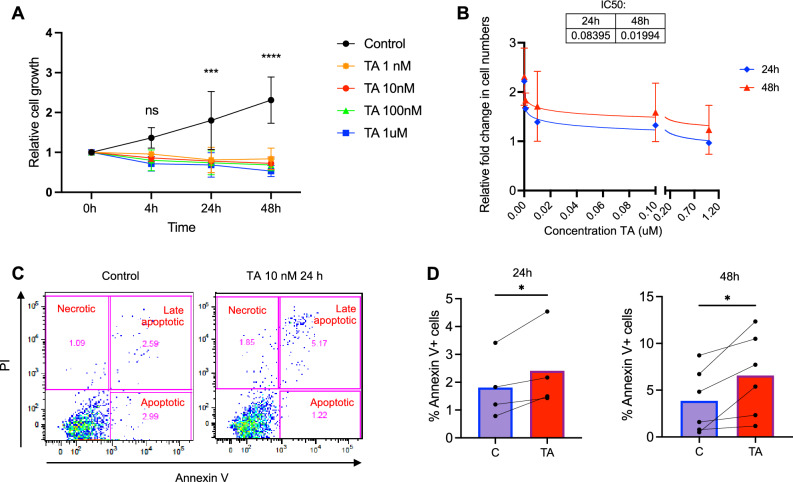
The effect of TA on MSC viability.

We found that TA induced a time and dose dependent significant inhibition of cell proliferation (Fig. 2A). The IC50 was 80 nM at 24 h and 19 nM at 48 h (Fig. 2B). There was an increase in apoptotic cell fraction after TA treatment relative to that in the untreated cells (Fig. 2C), demonstrating that TA exposure induced MSC apoptosis ex vivo (Fig. 2D)*.*

### TA impaired colony forming capacity of BM MSCs

**Figure 3:**
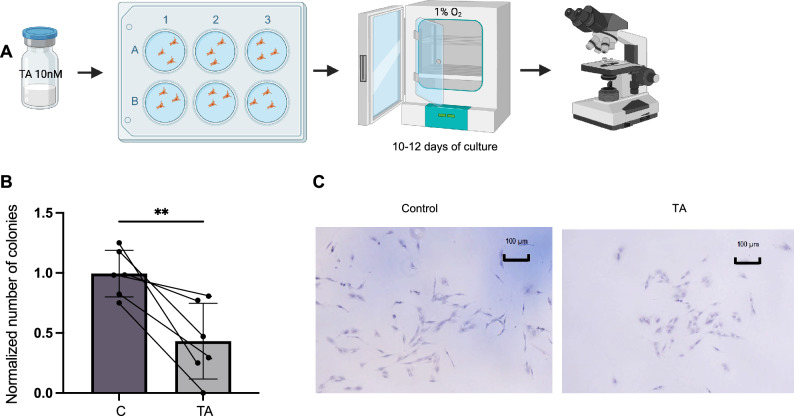
The effect of TA on MSC maintenance.

Fibroblast colony forming capacity is considered a functional feature of MSCs. The number of CFU-Fs reflects the functionally defined MSCs. To determine the effect of TA on CFU-F activity, we performed CFU-F assay of the MSCs after 24 h- treatment with TA (10 nM) at very low density (200 cells/well) in 12-or 6-well plates (Fig. 3A). Interestingly, CFU-F frequency was significantly reduced in the MSCs treated with TA (Fig. 3B). In addition, morphology of TA-treated CFU-Fs were altered where cells appeared smaller in size than that derived from the untreated MSCs (Fig. 3C), altogether indicating that TA exposure impaired the maintenance of MSCs.

### TA treatment cause increased adipocyte differentiation potential while impairing chondrogenenesis

**Figure 4:**
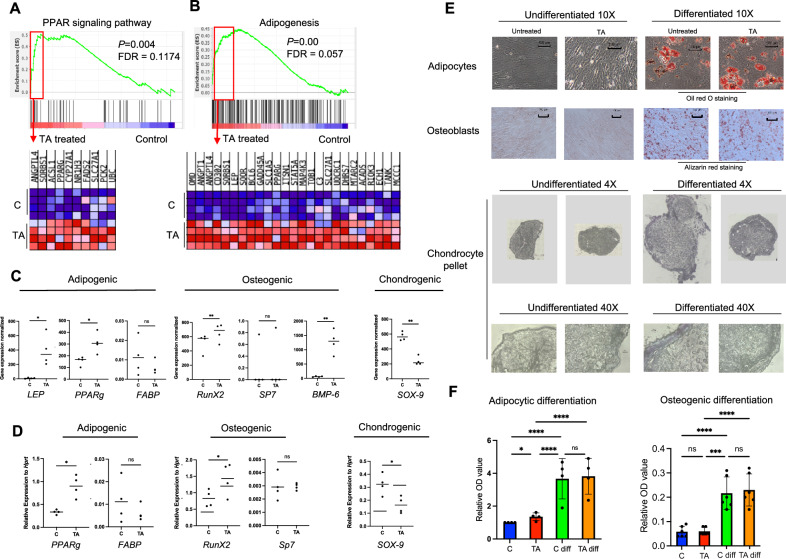
The effect of TA on MSC differentiation.

Multilineage differentiation potency is one of the characteristics of BM MSCs and highly associated with tissue regeneration capacity of the cells. In our previous study using a murine embryonic fibroblast (C3H10T1/2) cell line, TA had a negative impact on tenocytic differentiation while adipocytic differentiation was promoted^12^. However, it is not clear whether TA will induce similar effects on adult human BM MSCs. To assess this, we analyzed gene expression of lineage-associated genes as well as multilineage differentiation assays. RNA sequencing of the MSCs revealed upregulation of the gene sets associated with peroxisome proliferator-activated receptors (*PPAR*) signaling and adipogenesis in the MSCs exposed to TA 10 nM for 24 h (Fig. 4A-B) pointing to a possible spontaneous adipocyte differentiation of the MSCs after TA treatment. This includes *PPAR*gamma *(PPAR*γ*)*, the master switch of adipogenesis^21,22^ and *LEPTIN (LEP),* a hormone produced by adipocytes (Fig. 4C). This spontaneous differentiation was confirmed by significantly increased oil red O staining which was quantified photometrically (Fig. 4F). However, there was no significant increase of *FATTY ACID BINDING PROTEIN 4* (*FABP4*), a late adipocytic gene expression marker (Fig. 4C). These finding were consistent with PCR-findings, showing significantly increased *PPAR*γ, however no significant output of *FABP4* (Fig. 4D). There was no significant change in adipocyte differentiation after stimulating the treated cells with adipogenic induction media (Fig. 4E, first row), suggesting the adipogenic differentiation potential may not differ dramatically. It is also possible that the differentiation induction media was too strong to reveal any potential difference in the differentiation, since all the MSCs might be forced to differentiate into adipocytes after 3 weeks of induction.

The effect of TA stimulation on the osteoblast differentiation potential was not functional as we did not observe significant difference in calcium deposition in the differentiated osteoblasts between the TA-treated and untreated MSCs (Fig. 4D, second row). However, RNA sequencing indicated upregulation of transcripts of *Runt-related transcription factor X (RunX2)*, a key regulatory gene involved in driving MSCs toward osteogenic fate and *Bone Morphogenetic Protein 6 (BMP-6)*, an osteoinducing member of the Transforming Growth Factor-beta superfamily (*TGF-β*) (Fig. 4C). However, *Sp7*, a bone specific gene expression marker, important for maturing osteoblasts and bone homeostasis, showed no significant increased expression neither by gene expression quantification (Fig. 4D), or by Q-PCR assay (Fig. 4E). Overall, the data suggest that TA may have a molecular effect on osteogenic capacity of MSCs, however this was not verified in the functional assay.

Consistent with our previous observation on mouse fibroblasts, we found that TA impaired chondrocyte differentiation of the MSCs, as shown by smaller pellet and less proteoglycan staining identified by toluidine blue in the 3D micromass cell pellet compared to that from the untreated MSCs (Fig. 4E, third and fourth row). This notion was further consistent with the reduced transcripts of *Sex-determining region Y-box 9 (SOX-9)*, a pivotal transcription factor for chondrogenesis^21^, is significantly reduced in cells exposed to TA, supporting that TA may have a negative effect on chondrogenesis (Fig. 4C) and verified with PCR (Fig. 4D).

### The impact of TA on transcriptomic profile of MSCs

**Figure 5:**
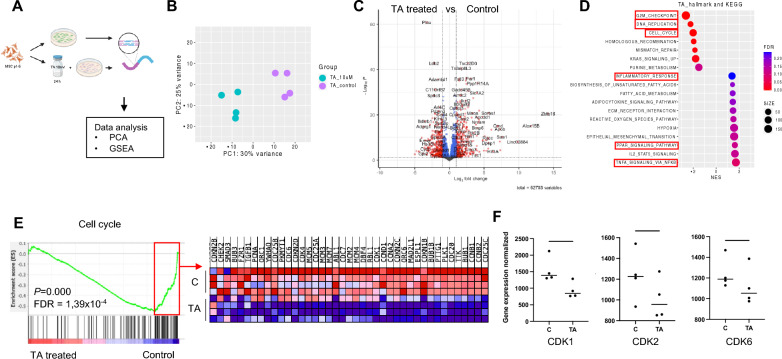
The impact of TA on MSC transcriptomic profile.

To further reveal molecular changes in the MSCs after TA treatment, we analyzed the RNA sequencing data using Principal Components Analysis (PCA) and Gene Set Enrichment Analysis (GSEA) (Fig. 5A). PCA (Fig. 5B) showed distinct gene expression profiles of the TA-treated MSCs from the control MSCs. Volcano plot (Fig. 5C) showed significantly altered genes in TA-exposed MSCs versus vehicle treated controls. The genes that were altered dramatically (*P *values < 0.05 and log_2_fold changes > 1) are highlighted in red in the volcano plot and in the Supplementary Table 1.

Further analysis with GSEA revealed downregulation of gene sets related to G2M checkpoint, DNA replication and cell cycle (Fig. 5D). The gene alterations are in keeping with our finding of impaired MSC proliferation associated with induced apoptosis (Fig. 2). Specifically, *cyclin dependent kinase (CDK)* 1 and 2, cell cycle regulators^23^, were reduced in MSCs subjected to TA (Fig. 5E–F). These data support that TA has a detrimental effect on cell regulation and proliferation even at low concentrations and for short time exposure.

### The effect of TA on inflammatory response of MSCs

**Figure 6:**
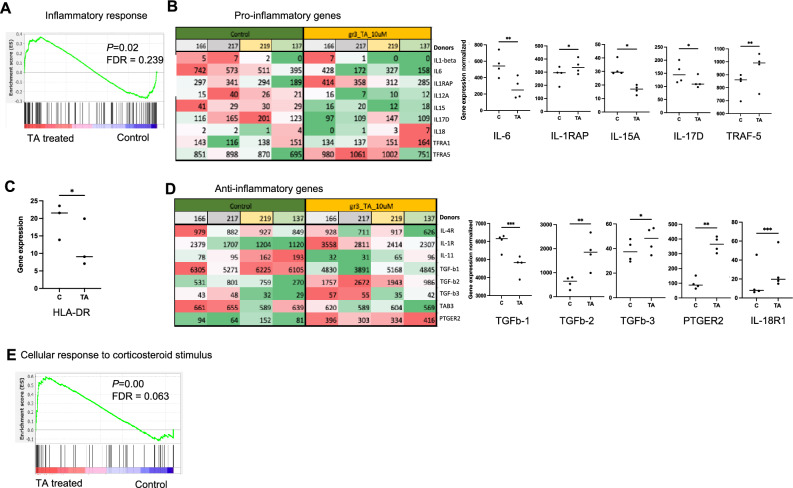
The effect of TA on MSC inflammation.

GSEA revealed an overall upregulation of inflammatory response (Fig. 6A). Further analysis of pro- and anti-inflammatory genes showed a downregulation of pro-inflammatory genes in the MSCs exposed to TA, supporting the anticipated clinical anti-inflammatory effect of TA (Fig. 6B), rendering less activation of MSCs, altering the MSC immunomodulating properties. This is supported by downregulation of *HLA-DR* (Fig. 6C) which can be activated upon inflammation^24^. Consistent with this, anti-inflammatory cytokines, such asprostaglandin E receptor 2 (*PTGER2*), Transforming Growth Factor beta 2 and 3 (*TGF-β2, TGF-β3*) were also upregulated. Interestingly and as expected, the cellular response to corticosteroid stimulus was enhanced after TA treatment (Fig. 6E), confirming the effect of glucocorticoid TA treatment on MSCs.

Of note, genes associated with epithelial-mesenchymal transition, fatty acid metabolism, reactive oxygen species and hypoxia were also upregulated (data not shown). These alterations may act together and lead to potential impact on the related cellular response and biological processes in the MSCs post TA exposure, e.g. oxidative stress in the local microenvironment and cell–cell/matrix interactions.

## Discussion

**Figure 7:**
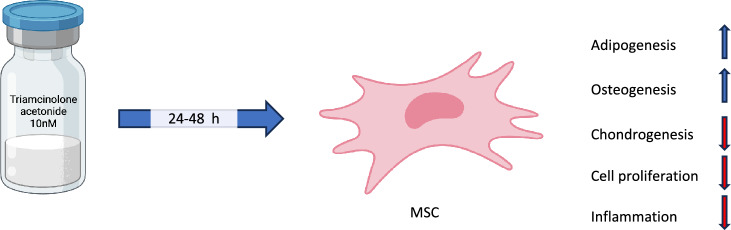
A summary showing hypothetic working mechanism of TA on MSCs.

In this study, we have explored the impact of TA on BM MSCs. Of note, our experiments were performed under hypoxic culture conditions, which is critical for maintaining MSC expansion and differentiation potential, mimicking the in vivo environment^25–28^. Here, we show that short term exposure (24 h) of human BM MSCs with TA impaired the maintenance and differentiation of MSC. Furthermore, TA treatment also altered inflammatory responses and downregulation of cell cycle related genes (Fig. 7).

We here have provided evidence for TA effects on MSCs and the underlying mechanisms, deteriorating proliferation both by apoptosis but also by directly affecting cell cycle progression, which has never been shown previously^11,29^. The effect on MSC self-renewal hits several key regulatory checkpoints such as DNA replication and cell cycle passage, resulting in impaired regeneration.

We also provide evidence for the primed adipocyte differentiation following exposure to TA, as clearly shown by RNA sequencing, showing upregulation of PPARγ. We found that TA upregulated Leptin, a hormone released by adipocytes, as well as an increased spontaneous adipogenesis in the MSCs subjected to TA. Thus, we speculate that longer exposure time and higher concentration of TA, which likely occurs in the clinical setting, may scew MSC fate toward adipogenic fate and further impair cell growth^30^.

There is a presumed inverse relationship between adipogenesis and osteoblastogenesis where PPARγ is considered the master key regulatory gene of adipogenesis and RunX2 the osteoblastic equivalent. The effect of glucocorticoids in different concentrations, physiologic or therapeutic, has been discussed by Han et al.^22^ where low physiological concentrations of dexamethasone promote osteogenesis and higher therapeutic concentrations induces osteopenia and osteoporosis, favoring osteoclast activity when administrated systemic^30^. Our work shows a simultaneous upregulation of adipogenic markers, PPARγ and osteogenic markers e.g., BMP-6 and RunX2. Nevertheless, the functional consequences appear to be minor as we did not observe any significant changes in osteogenic differentiation in vitro. The discrepancy between gene expression and osteoblast maturation may be due to the effective osteo-inductive media, overriding the early molecular priming of osteogenic lineage^31,32^.

The effect of TA on chondrogenesis is striking, both suppressing chondrogenesis on a molecular level, reducing expression of SOX9, the regulatory transcription factor of chondrogenic differentiation^33–35^, and producing a smaller cell pellet compared to control and affecting proteoglycan production negatively^36^. This could also be an effect of MSCs osteoblastic induction, downregulating and suppressing chondrogenesis, as RunX2 is involved in both early osteoblastogenesis and chondrogenesis^34^.

. It is well accepted that MSCs possess an immunomodulatory property through cell–cell interaction^37,38^ and that some degree of inflammation is mandatory for cell recruitment and maintaining the healing process^39^.

Our finding of upregulation of the anti-inflammation response by TA treatment is consistent with glucocorticoid therapeutic effect^40^. However, it is also possible that the anti-inflammatory cytokines may act on the MSCs via paracrine manner^41^, thus, MSCs become less activated, reflected in the reduced expression of HLA-DR in the TA-treated MSCs. This speculation is supported by improved MSC migration which was further increased by TA treatment (1uM)^42^.

Although this study was performed in a simulated hypoxic in vitro environment, which is more suitable for MSC expansion and maintenance^26^, the conditions may differ from the in vivo microenvironment in the joint during early injury-healing phases where inflammation is greatly elevated. The MSCs respond and transform to culture environment e.g. oxygen pressure, medium conditions and culture set-up, where in vitro cells are grown two-dimensionally, thus significantly differ from in vivo conditions in the bone marrow niche^38^. Explanted MSCs in vitro*,* spontaneously alter both secretomic profile as well as phenotypical appearance depending on seeding- density^43^ and to acquire more robust support for our hypothesis, having a more realistic and physiological model would be of interest. To mimic clinical treatment with TA, treating MSCs with TA for longer time or using an animal model could be performed as a next step.

In addition, the functional consequences of many dramatically altered inflammatory response genes have not been investigated. The protein expression and corresponding signaling pathway should be further explored in the future.

In summary, our data show that TA, already at low dose and short time exposure, has a potentially devastating effect on regeneration and healing, disturbing the immunological environment.

Altogether, our findings call for the extra consideration of the healing properties when aiming at relieving pain and restoring function in the synovial joint, securing our primary obligation; to never harm the patient.

### Supplementary Information

Supplementary Information 1.

Supplementary Information 2.

## Data Availability

The RNA data sequencing data are available at https://eur01.safelinks.protection.outlook.com/?url=https%3A%2F%2Fwww.ncbi.nlm.nih.gov%2Fgeo%2Fquery%2Facc.cgi%3Facc%3DGSE240387&data=05%7C01%7Chong.qian%40ki.se%7C6662a89d54e44986a62008dbd5f6dbee%7Cbff7eef1cf4b4f32be3da1dda043c05d%7C0%7C0%7C638339028459243422%7CUnknown%7CTWFpbGZsb3d8eyJWIjoiMC4wLjAwMDAiLCJQIjoiV2luMzIiLCJBTiI6Ik1haWwiLCJXVCI6Mn0%3D%7C3000%7C%7C%7C&sdata=Dvrnk1%2B1fBVHSdIp2N9LxoIK%2BhSsmDhtP%2FdkqUWb3pY%3D&reserved=0. Please use toke exmlqiqsttktnqv to be put in the box. The rest of data can be provided upon request by corresponding author.
