# Supplementary material for: The functional and molecular impact of triamcinolone acetonide on primary human bone marrow mesenchymal stem cells
Source: Sci Rep. 2023 Dec 8;13:21787. doi: 10.1038/s41598-023-48448-z (PMC10709330; doi:10.1038/s41598-023-48448-z)
Supplement: Supplementary file 2 — Supplementary Information 2. [file 41598_2023_48448_MOESM2_ESM.docx]

**Supplementary Table 2 List of antibodies**

Following flourochrome-conjugated antibodies were used to determine MSC purity:

CD45-PB BV421

CD44 APCCy7

CD31 PEcy7

CD105 APC

CD90 PEcy5

**Supplementary Table 3 List of TaqMan** **qPCR probe**

| **Gene** | **Assay ID** |
| --- | --- |
| *PPARγ* | Hs01115513_m1 |
| *FABP4* | Hs01086177_m1 |
| *Runx2* | Hs00231692_m1 |
| *Sp7* | Hs01866874_m1 |
| *Sox9* | Hs01001343_g1 |
| *Hprt* | Hs99999909_m1 |
